# Clinical use and complications of percutaneous cystostomy pigtail catheters in 25 cats

**DOI:** 10.1177/1098612X221080902

**Published:** 2022-04-01

**Authors:** Genziana Nurra, Charlotte Howes, Guillaume Chanoit, Lee Meakin, Kevin Parsons, Ed Friend

**Affiliations:** 1 Small Animal Referral Hospital Langford, University of Bristol, Bristol, UK; 2 Soft Tissue Service, University of Bristol, Bristol, UK; 3 Orthopaedic Service, University of Bristol, Bristol, UK

**Keywords:** Feline lower urinary tract disease, urinary tract trauma, neurogenic, urinary disease, pigtail catheter, urinary diversion

## Abstract

**Objectives:**

The aims of this study were to describe the indications for percutaneous pigtail catheter placement in cats requiring urine diversion, and to report the associated intra- and postoperative complications.

**Methods:**

The medical records of cats that underwent percutaneous pigtail catheter placement for urine diversion between January 2011 and May 2021 were retrospectively reviewed.

**Results:**

Twenty-five cats were included. Indications for pigtail catheter placement were medical management of obstructive urinary tract disease (n = 12), urinary tract damage after traumatic injury (n = 8) and neurological bladder dysfunction (n = 5). Catheters were in place for a median time of 8.28 days (range 3–27), and the duration of the catheter placement was not different between the medical, traumatic and neurological groups. Ten cats (40%) developed pigtail catheter complications including dislodgement, urine leakage, urinary tract infection and bladder rupture. The majority of complications were easily resolved and did not require surgical intervention.

**Conclusions and relevance:**

The results suggest that percutaneous pigtail catheter placement can facilitate urine diversion in both the emergency setting and in the long-term management of urine retention without many complications.

## Introduction

Temporary urinary diversion is indicated in patients with functional or mechanical obstruction of the bladder or urethra leading to excessive urine retention. The most common indications include urinary outflow obstruction secondary to feline lower urinary tract disease (FLUTD), uroliths, urethral stricture, urinary tract trauma and neurogenic disease resulting in bladder detrusor atony.^1–3^ Temporary urinary diversion can be performed in an emergency situation for traumatic lesions of the urinary tract or as a part of the medical management to stabilise patients with metabolic abnormalities associated with urinary tract obstruction. Patients with neurogenic abnormalities, in which the bladder is difficult to manually express and are non-responsive to medical therapy, may also be candidates for diversion of urine until the bladder is restored to function.

Retrograde urethral catheterisation and open or minimally invasive surgical cystostomy tube insertion^4,5^ are historically the main methods described for short- or long-term urinary diversion in cats and dogs. However, these options are not possible in every case: unsuccessful resolution of mechanical obstruction or the presence of urethral stricture may not allow the insertion of a retrograde indwelling catheter. Urethral rupture is also a well-documented complication secondary to traumatic urethral catheterisation.^6,7^ Recurrent or persistent urinary tract infection is another complication associated with indwelling urethral catheters or cystostomy tubes.^2,5,8,9^ In a retrospective study conducted in 76 dogs and cats, complications occurring after cystostomy tube placement were encountered in half of the patients and included dislodgement and removal in the majority of cases, and irritation and leakage around the tube site in the minority of cases.^6^ Pigtail cystostomy tube placement is a relatively novel method to divert urine. This technique, in particular, can be helpful in the emergency setting because of the advantage that it does not require general anaesthesia and an open surgical approach.

Patients that present with mechanical or functional urinary tract obstruction and secondary urinary retention may have perfusion abnormalities and marked metabolic derangements secondary to post-renal azotaemia and may tolerate general anaesthesia poorly. Traditionally, cystostomy tube placement has been the most recognised urinary diversion technique.2 However, to allow adequate surgical exposure and manipulation of the bladder, this procedure requires a ventral median celiotomy. In contrast, the pigtail catheter is a quick and relatively technically undemanding technique, which minimises surgical time and allows easy access to the distended urinary bladder. The major advantage is that the placement of pigtail catheters does not require an open surgical approach: the patient does not need to be moved to a sterile theatre, and the procedure can be performed either in the imaging or preparation room, thus minimising the anaesthesia time.

The pigtail catheter is an alternative option that allows patient stabilisation when urethral obstruction cannot be easily relieved by retrograde hydropulsion and urethral catheterisation. In animals with traumatic urinary tract injury, the use of a pigtail catheter allows long-term urinary drainage and improves the comfort of the cats during the perioperative period. Pigtail catheters can be also placed to facilitate urinary drainage in animals with neurological damage secondary to sacro-coccygeal injuries and with bladder detrusor atony. This procedure minimises the stress of patients that require long-term manual decompression of the bladder until neurological function can be restored.

The use of the pigtail catheter has been reported in dogs^10,11^ and humans^12,13^ but has not been fully reported in cats; a small case series describing the use and complications of percutaneous loop cystostomy catheters in cats has been published.^14^

Three cats with urethral obstruction secondary to feline idiopathic cystitis were included in this study. The use of a locking loop-style catheter was associated with a high risk of complications. The complications were encountered within 24 h in all three cats and included dislodgement from the bladder with leaking of urine and snagging of the omentum by the locking loop mechanism. In all cats, surgical removal of the catheter was required. Thus, information on indications for the use of percutaneous cystostomy pigtail catheters in a larger number of cats, and complications associated with their use is lacking.

The aims of this study were, therefore, to describe the potential indications for percutaneous pigtail catheter use in cats and the complications associated with their use.

## Materials and methods

The medical records of cats treated at the University of Bristol Veterinary Teaching Hospital (Langford Vets) were retrospectively reviewed to identify patients that underwent pigtail catheter placement between January 2011 and May 2021.

### Data collection

Information retrieved from the medical records of the cases included signalment, indication for pigtail catheter placement, diagnosis, length of time the pigtail catheter was in place, periprocedural complications and the results of urine bacterial culture. Duration of catheter use was classified as short term if the catheter was maintained for ⩽ 7 days and long term if it was maintained for > 7 days. Pigtail catheter complications were classified as minor if they resolved or responded to medical treatment without the necessity of surgical intervention. Catheter complications were classified as major if they required catheter replacement or surgical intervention.

### Surgical procedure

**Figure 1:**
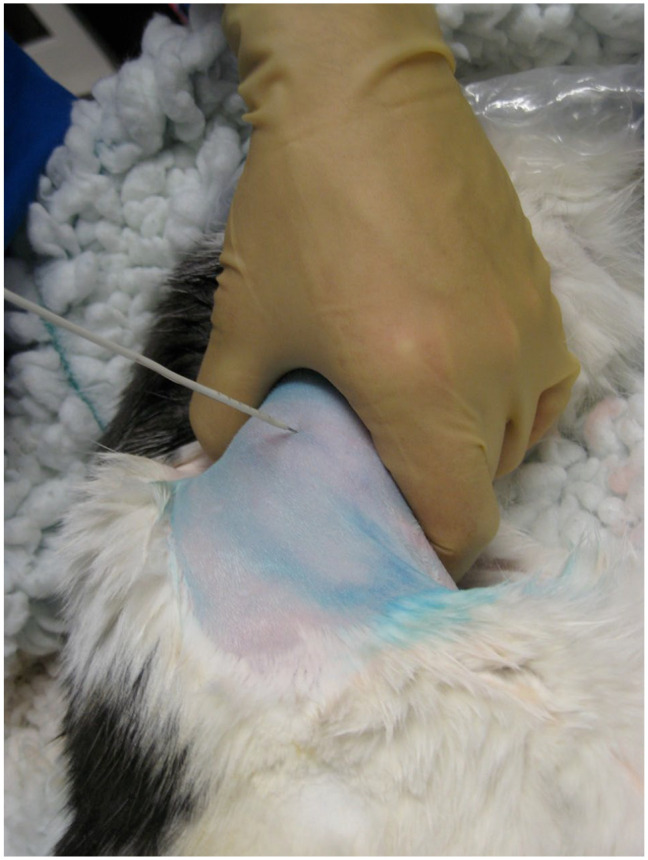
The cat was positioned in left lateral recumbency, after standard aseptic preparation of the ventrolateral abdomen. A stab incision was previously made to the inguinal region. The image shows the advancement of the catheter/hollow trochar/needle stylet system into the bladder

**Figure 2:**
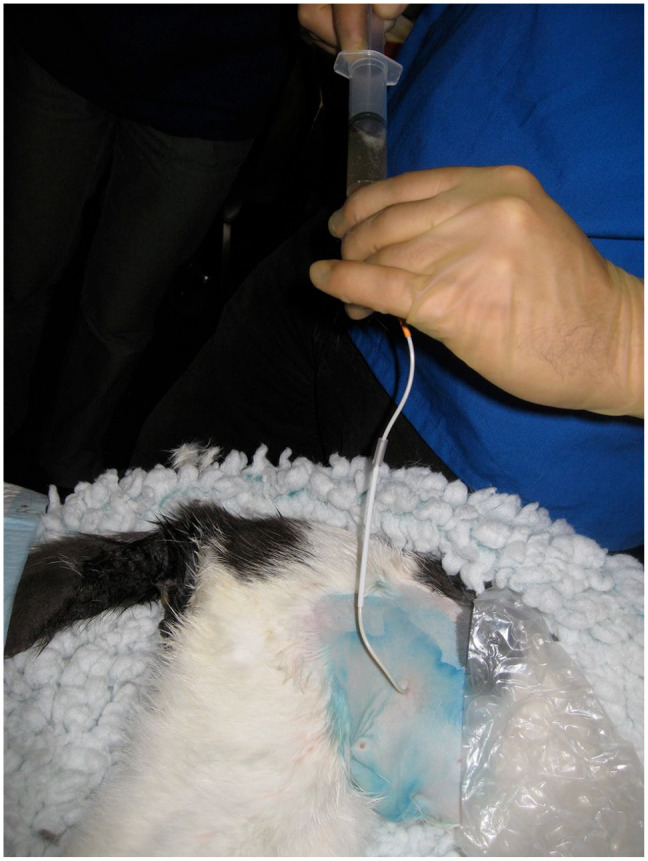
To confirm the location of the pigtail catheter in the urinary bladder a syringe was attached and urine was aspirated

**Figure 3:**
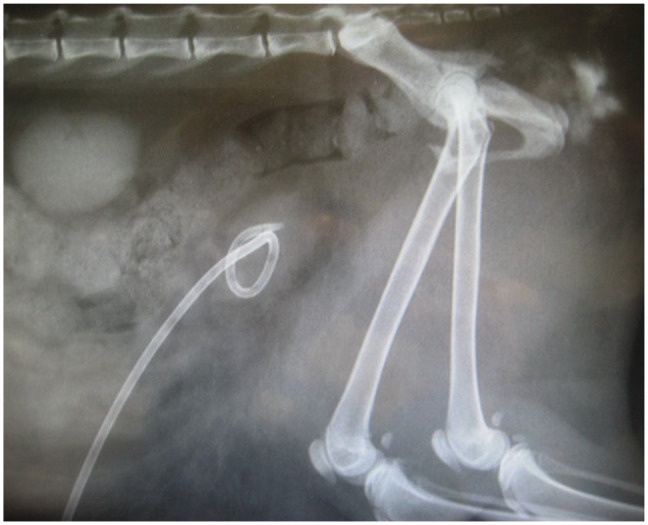
Lateral radiograph of the abdomen showing the pigtail catheter located in the bladder

The majority of the pigtail catheters were placed under general anaesthesia or sedation. This decision was case dependent and mostly influenced by the need of concurrent imaging or surgical treatment. The analgesia protocol was tailored to each patient according to the concurrent injury, but no specific analgesia was required for pigtail catheter placement. The catheter was placed according to the manufacturer’s guidelines (Infinity Medical) and the size used ranged from 6 F to 8 F, based on availability. Patients were positioned in lateral recumbency, and the lateral abdomen was widely clipped and aseptically prepped with gluconate chlorhexidine. In the majority of cases, the patients were not draped, in order to facilitate the bladder palpation. The bladder was palpated and stabilised (laterally towards the aseptically prepared abdominal wall) with the non-dominant hand (Figure 1). A superficial stab incision was performed in the skin overlying the bladder in the caudal abdominal region with a number 11 scalpel blade. The entire device containing the straightener, locking mechanism, puncture needle and stylet was advanced percutaneously until the device was seated in the urinary bladder. The stylet was then removed and a 5 ml syringe attached and aspirated to confirm the presence of urine (Figure 2). The pigtail catheter was further advanced into the bladder. The catheter locking loop mechanism was activated by pulling and securing the locking string. The pigtail catheter was attached to a sterile urinary bag and secured to the body wall with finger trap suture at the incision site. Radiographs of the abdomen were obtained to confirm the presence of the cystotomy pigtail catheter in the bladder (Figure 3). Tube management consisted of monitoring urinary output and cleaning of the stoma site. The stoma site was cleaned with diluted chlorhexidine twice daily and protected with a sterile adhesive drape (Allevyn), which was applied over the catheter site to prevent contamination. Analgesia and antimicrobial therapy were tailored to the individual patient. The indication of prophylactic antibiotics was based on the antimicrobial use guidelines for the treatment of urinary tract disease.15

Antibiotics were given to patients that experienced concurrent urinary disease, such as a urinary tract infection (UTI) and urolithiasis. The presence of a UTI was diagnosed by cytology followed by culture and sensitivity testing. The first-line antibiotic was initiated and potentially changed based on the microbiology and sensitivity results.

**Figure 4:**
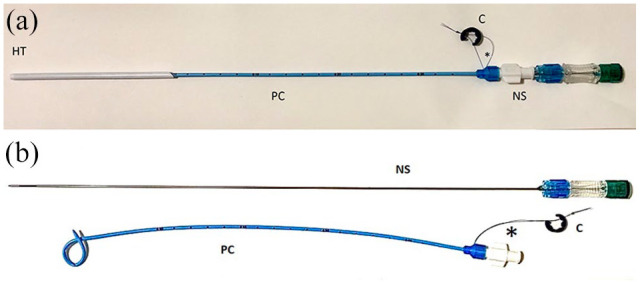
Parts of the pigtail catheter. (a) Assembled. (b) Partially assembled to demonstrate how the components fit together. Once in place in the urinary bladder, the suture pictured below the hub of the catheter is pulled to ensure that the pigtail has been formed. *Suture to lock loop. C = collar; NS = needle stylet; HT = hollow trocar; PC = pigtail locking-loop catheter

The pigtail catheter was removed 5 days after placement, unless it became dislodged. The plastic collar, which secures the locking loop mechanism (Figure 4), was released and the catheter was gently removed. Removal of the pigtail catheter did not required sedation.

## Results

### Signalment

Twenty-five cats met the inclusion criteria. The included breeds were domestic shorthair (n = 17), Persian (n = 2), British Shorthair (n = 1), Ragdoll (n = 1), Bengal (n = 1), Birman (n = 1), Siberian (n = 1) and Ragamuffin (n = 1). The median age was 49.24 months (range 8–204) and mean body weight was 4.27 kg (range 2.1–6.6). Twenty-three of the 25 (92%) cats were male (two entire and 21 neutered), and two cats were female (both entire). Urine collected from the urinary bladder was submitted for culture and sensitivity in 11/25 cats

### Indications for pigtail placement

For 12/25 cats (48%) the pigtail catheter was placed as part of medical management for urethral obstruction (secondary to uroliths n = 5, secondary to obstructive feline idiopathic urinary tract disease n = 7). Eight of the 25 cats (32%) had pigtail catheter placement for urinary tract dysfunction or rupture secondary to trauma (iatrogenic urethral tear n = 2, pelvis fracture n = 3, spinal fracture n = 1, urethral transection n = 2). Five of the 25 cats (20%) required pigtail catheter placement for bladder management secondary to neurological dysfunction (sacrococcygeal luxation n = 3, bladder detrusor atony n = 2) (Table 1).

**Table 1:** Demographic data and summary of indications for 10/25 cats with complications related to the pigtail catheter placement

Breed	Age (months)	Sex	Group	Indication	Diagnosis	Total duration of catheterisation (days)	Minor complications	Major complications
DSH	11	MN	Medical	UO	Urolith	9	Urine leakage + UTI	
DSH	59.9	MN	Medical	UO	Urolith	5	Pyrexia	
Persian	8	MN	Medical	UO	FLUTD	27	UTI	
DSH	59.9	MN	Medical	UO	FLUTD	5		Bladder rupture
DLH	24	MN	Medical	UO	FLUTD	6	Pyrexia	
DSH	12	MN	Medical	UO	FLUTD	12	Pigtail catheter dislodgement + haematuria	
DSH	67	MN	Trauma	UO	FLUTD	5	Stranguria	
Siberian	36	MN	Trauma	RTA	Spinal fracture	20	UTI	Pigtail catheter dislodgement with uroperitoneum
DSH	36	MN	Neurological	RTA	Sacrococcygeal luxation	9	UTI	
Bengal	19	FE	Neurological	RTA	Sacrococcygeal luxation	7	Urine leakage	

DSH = domestic shorthair; MN = male neutered, UO = urethral obstruction, UTI = urinary tract infection, DLH = domestic longhair; FLUTD = feline lower urinary tract disease; RTA = road traffic accident; FE = female entire

### Duration of the pigtail use

Duration of the pigtail catheter use was classified as short term (ie, ⩽ 7 days) in 13 cats (obstructive urolithiasis n = 4; obstructive feline idiopathic cystitis n = 4; traumatic urethral rupture n = 1; pelvis fracture n = 2; sacrococcygeal luxation n = 2). Duration of the pigtail catheter was classified as long term (ie, > 7 days) in the remaining 12 cats. For all cats, the median time the pigtail catheters were in place was 8.72 days (range 3–27). The median time pigtail catheters were in place for cats with medical management was 8.71 days (range 2–27), for cats with urinary tract trauma 7.5 days (range 2–20) and for cats with neurological dysfunction 8.5 days (range 6–11).

### Complications

Ten of the 25 cats (40%) had complications related to the pigtail catheter placement. Three of the 25 (12%) had more than one complication. Two cats had major complications associated with the pigtail catheter, which consisted of bladder rupture in one cat and dislodgement of the catheter from the bladder with secondary uroperitoneum in the second cat. In the cat in which bladder rupture occurred, the bladder was surgically repaired, and the pigtail catheter was replaced. In the cat in which the pigtail catheter displaced from the bladder, the pigtail was initially further inserted into the bladder and monitored for 24 h and subsequently replaced with a permanent cystotomy tube. In one cat, dislodgement of the pigtail catheter and secondary haematuria occurred. Urine culture was negative, and the catheter was removed.

Five cats had minor complications associated with the pigtail catheter, which included pyrexia (n = 2), persistent stranguria (n = 1), UTI (n = 4) and urine leakage around the tube (n = 1). In one of the two cats that developed pyrexia, a purulent discharge was also identified around the peristomal area. Both patients with pyrexia were started on prophylactic antimicrobial therapy while the urine culture results were pending. In the cat with spinal fracture, UTI occurred 19 days after the pigtail catheter placement and was diagnosed based on culture and sensitivity urine analysis. Antimicrobial therapy was initiated for this patient. One cat developed urine leakage around the catheter and an ultrasound scan of the abdomen detected a moderate amount of peritoneal effusion suggestive of peritonitis. Fluid analysis was consistent with a modified transudate without evidence of uroperitoneum (Table 1).

## Discussion

The present study describes the indications and the complications associated with the use of a percutaneous pigtail cystostomy catheter in cats.

To the authors’ knowledge this is the first study that reports complications associated with the use of percutaneous pigtail cystostomy catheters in cats. Ten of the 25 (40%) cats in the present study developed complications related to the pigtail cystotomy catheter; therefore, potential complications should be discussed with owners prior to pigtail catheter placement.

The complication rate we reported for pigtail catheters in this study was slightly lower compared with previous studies where the pigtail was surgically placed. Previous studies, conducted in dogs and cats, evaluating outcomes for cystotomy tube placement reported 49% and 53% complication rates for cystotomy tubes inserted through celiotomy and a minimally invasive inguinal approach, respectively.^6,15^ Although complications occurred frequently, these were mainly minor, which is similar to the findings described in those studies,^6,8,16^ where major complications were less commonly reported. Most complications were self-limiting and did not interfere with the catheter use or affect the animal’s quality of life. Data on pigtail catheters in cats have not been previously reported in the literature and therefore comparisons are difficult; nevertheless, the complication rate we reported appears lower than what has been previously observed.

Iatrogenic bladder rupture was documented in one cat. Dislodgement of the pigtail catheter was encountered in two cats. Similar complications were observed with the use of a percutaneous locking loop-style cystostomy catheter, in which all three included cats had partial dislodgement from the bladder and subsequent surgical exploration for catheter removal.^14^ One explanation may be that the catheter was not completely advanced into the bladder, preventing the pigtail from opening completely.

UTI and haematuria were also other complications encountered in this study. This was not unexpected as it is well known that recurrent or persistent urinary tract infection and haematuria are also common complications described with the use of indwelling catheters and percutaneous cystostomy.^2,6,9^ UTI is most likely to be related to residual urine in the bladder and the formation of a biofilm on the tube.^17,18^

It is possible that the level of experience of the clinician influenced the rate of complications seen. However, this information was not available from the clinical records. Another limitation of this study is the lack of information in the anaesthetic records about the time required to place the percutaneous pigtail catheter, as the anaesthesia time also included the diagnostic investigations, which have been performed beforehand.

## Conclusions

The urinary diversion technique reported herein has numerous advantages. It can be considered for cats with various conditions related to problems with urine outﬂow, both in emergency situations and in the long-term management of urinary bladder decompression. It is a minimally invasive procedure and allows for easy and quick placement. However, almost half of the cats included in the study developed complications and therefore potential complications should be discussed with owners prior to catheter placement.
